# Modeling hydro, nuclear, and renewable electricity generation in India: An atom search optimization-based EEMD-DBSCAN framework and explainable AI

**DOI:** 10.1016/j.heliyon.2023.e23434

**Published:** 2023-12-10

**Authors:** Indranil Ghosh, Esteban Alfaro-Cortés, Matías Gámez, Noelia García-Rubio

**Affiliations:** a IT & Analytics Area, Institute of Management Technology, Hyderabad, Telangana, India; b Quantitative Methods and Socio-economic Development Group, Institute for Regional Development (IDR), University of Castilla-La Mancha (UCLM), Albacete, Spain; c Faculty of Economics and Business Administration, University of Castilla-La Mancha (UCLM), Albacete, Spain

**Keywords:** Clean electricity, Empirical ensemble mode decomposition, Industrial growth, Atom search optimization, Explainable artificial intelligence

## Abstract

**Background and objective:**

Tracking clean electricity generation in developing economies is highly challenging owing to the influence of turbulent external factors. Clean electricity is a significant enabler of striving toward environmental sustainability. In this research, we aim to model hydro, nuclear, and renewable electricity generation in India through applied predictive modeling. We also strive to uncover the influence of the critical determinants responsible for clean electricity growth.

**Methodology:**

We propose a granular predictive framework comprising ensemble empirical mode decomposition, clustering applications in spatial data based on density, including noise, and atom search optimization-based novel optimization methodology to predict absolute figures of clean energy generation. The framework uses a series of socio-economic factors reflecting household demand and industrial growth in India as explanatory variables.

**Results:**

The rigorous scrutiny of the predictive framework specifies hydro electricity generation is relatively more predictable during the time horizon influenced by the COVID-19 pandemic. The deployment of dedicated explainable artificial intelligence (AI) tools suggests an increased adoption of clean electricity in selected industrial sectors in India, which broadly governs the evolutionary pattern.

**Conclusion:**

The underlying research is the first of its kind to fathom the daily temporal dynamics of clean electricity generation in the Indian context. Consideration of three distinct clean electricity sources during highly volatile time regimes underscores the contribution of the work. The predictive framework survives a stringent performance check, which justifies the robustness of the same. Demand in different industrial sectors in India profoundly influences the growth toward clean electricity.

## 1 Introduction

Renewable energy sources are vital for a sustainable low-carbon society [[1], [2], [3]]. The power in the form of electricity manifests the status of household and industrial activities of a nation [4]. The nexus of electricity supply and demand is largely governed by setting reasonable electricity prices. The predictability of electricity prices for boosting the market has been introspected thoroughly in the literature [5,6]. Nevertheless, it is equally essential to delve into the dynamics of the electricity generation process to anticipate the futuristic movements of energy reserve, excess power, growth of the business, etc. A precise prediction of electricity generation from renewable sources explicitly can be translated into practical implications for tracking the overall economic affairs [[7], [8], [9]]. An accurate forecast of electricity generation can assist policymakers in managing the energy mix to ensure grid stability, security of supply, etc., by adequately integrating renewable energy sources.

Mining the inherent pattern generation and energy consumption has been chiefly confined at the micro level, covering buildings, commercial processes, vehicles, etc. The said research category needs to be revised to measure the appetite for clean energy intake in nations in the context of de-carbonization. On the other hand, as stated, electricity price forecasting has seen considerable traction in literature [[10], [11], [12], [13]], predominantly owing to thwarting the supply-demand vagary in extreme weather conditions, geopolitical conflicts, etc. The influence of renewables on electricity price prediction has also been explored [14,15]. Renewable electricity generation is linked to societal implications, too [16]. Crozier and Baker [17] exemplified the utility of renewable electricity in cross-border interconnection in terms of power trading. Renewable resources have been marked to be necessary for electricity mix transformation in Germany [18]. Kan et al. [19] elicited the utility of hydro power for low-cost renewable power generation. Khosravi et al. [20] highlighted how investment in nuclear power could help stabilize the future electricity prices in Finland, enabling the transition away from coal by 2029. The effectiveness of hydro and nuclear assets in generating sustainable power is widely acknowledged in the literature [[21], [22], [23], [24]]. The paucity of research to model clean electricity generation at the macro scale is amply apparent. Considering the disruptive impact of the COVID-19 pandemic on production and business, and most recently, the unprecedented military conflict between Russia-Ukraine, it is arduous and practically necessary to closely introspect the pattern and dependence structure of clean energy generation from different renewable sources. The lack of substantial research in developing a predictive analytics framework is a significant roadblock to comprehending the interplay of clean energy generation with socio-economic factors.

The current work endeavors to fill the research void by creating a novel modeling framework to predict and decode clean energy generation patterns at the country level. The contribution of this research can be divided into three main categories. Firstly, we strive to predict India's daily hydro, nuclear, and renewable electricity generation, explicitly covering the COVID-19 pandemic timeline. Predicting the structure of electrical power generation of selected resources is the first of its kind compared to the existing literature. Secondly, the current work profoundly delves into the dependence of the chosen electricity generation components on a series of socio-economic factors in the Indian context. Activities of commoners captured through Google search volume index data are used as determinants of electricity generation, which act as representations of demand. Additionally, the financial outlook of several sectoral indices is used as explanatory variables to incorporate the demand for electricity for different industrial purposes. The aforesaid combination of explanatory variables effectively accounts for the influence of both household and industrial demand on electricity generation. Thirdly, we also contribute to the methodology front by designing a hybrid granular predictive structure and vis-a-vis deploying state-of-the-art explainable artificial intelligence tools are employed to interpret how selected features influence predictions, facilitating the derivation of significant implications.

The methodological framework propounds a granular predictive structure driven by decomposition to achieve the goals. The daily time observations of raw electricity generation in India from hydro, nuclear, and renewable resources are initially disentangled into granular sub-series using the ensemble empirical mode decomposition (EEMD) procedure. Afterward, the sub-series are clustered into high and low-frequency counterparts. The Hurst exponent and fuzzy entropy estimates of the respective sub-series are considered for clustering. To perform the time series clustering, the density-based spatial clustering of applications with noise (DBSCAN) has been adopted. Subsequently, predictive exercises are carried out on clustered high and low-frequency sub-series separately to yield component-wise predictions, aggregating to determine the final predictions. We design an ensemble of ensembles (EoE) type predictive structures infusing random forest (RF), bagging, gradient boosting (GB), and atom search optimization (ASO) in an optimization setup. Breaking down complex time series data into high and low-frequency segments has proven beneficial for forecasting volatile data. However, our novel predictive framework stands out due to its seamless integration of diverse tools, ensuring exceptionally accurate predictions. Rigorous numerical and statistical tests validate its superiority over benchmark models. Additionally, we derive crucial insights into how specific socio-economic factors impact electricity generation. We utilize explainable AI methods to uncover both global and local dependency structures. Using permutation feature evaluation, we pinpoint key contributors to clean energy generation in India. Then, accumulation local effect plots reveal the influence patterns of individual features, aiding strategic decision-making for managing hydro, renewable, and nuclear energy generation. Finally, the local interpretable model-agnostic explanations (LIME) framework offers insights into the prediction process at a local level. The deployment of XAI methodologies on top of the proposed granular predictive structure underscores the underlying research's effort to offer practical insights apart from the theoretical contributions.

The subsequent sectioning of this article unfolds as follows: Section 2 delineates related literature, aiming to identify research gaps and contextualize this work. Section 3 meticulously details the research methodology, describing individual tools and the procedural flow. Section 4 focuses on discussing data sources and the statistical properties of variables. Detailed results and comprehensive analyses are presented in Section 5. Section 6 critically examines the implications of the findings and proposes avenues for future research. Finally, Section 7 encapsulates the manuscript's conclusion.

## 2 Literature review

Tracking and predicting the electricity generation process has predominantly been carried out by analyzing the impact of different process parameters, climate factors, and physical microstructural properties [25]. Alternatively, using past historical records either as raw explanatory features or technical indicators for predicting electricity and energy generation patterns has been documented, too [26]. Implications manifested in the form of empirical research on the properties of renewable electricity have been highlighted in the literature. Sravan et al. [27] pointed out that Indian electricity consumption did not influence renewable electricity production. It was found that natural resource rents and greenhouse gas emissions affected renewable electricity generation positively. We summarize the research trend of clean electricity consumption and its impact on holistic development in developing economies in Table 1 to properly position the underlying work.

**Table 1:** Pertinent literature.

Authors	Endeavor and Methodology	Location
Tiwari et al. [28]	Confirmed the correlation between electricity consumption and economic growth using the Granger causality test within a vector autoregression framework	India
Liu et al. [29]	Propounded a quantum genetic algorithm-inspired fractional grey polynomial model to accurately estimate the electricity consumption	India and China
Rajkumari [30]	Explored the interplay between electricity consumption and economic reforms through Granger causality assessment and forecasted electricity demand using the Holt-Winters smoothening method	India
Wassie and Ahlgreen [31]	Delved into the influence of household size and configuration, income distribution, load pattern, etc., on solar electricity consumption using statistical modelling.	East Africa
Xu et al. [32]	Expounded on the strong direct and indirect nexus between clean electricity growth, ecological awareness and economic welfare using structural equal modelling	China
Abbas et al. [33]	Investigated how green finance, environmental tax policies, and geopolitical risk factors contribute to shaping investments within the renewable electricity ecosystem	China
Casati et al. [34]	Created a social clean energy access index aimed at assessing the societal effects of clean electricity and determining optimal countries for investing in renewable energy infrastructure	Sub-Saharan Africa
Espoir et al. [35]	Studied the influence of renewable electricity consumption on broader economic and financial growth employing both linear regression and kernel-based regularized least square models	Africa

The aforesaid literature clearly points out the close interlinkage among clean electricity generation, economic development, and societal reforms. Hence, the selection of the pertinent explanatory features in the Indian context will be critical to predict hydro, nuclear, and renewable electricity generation precisely. Also, the literature above primarily considers one specific source of clean electricity. We aim to extend the same by selecting three different sources of clean electricity generation for comprehensive modeling. We now enunciate the critical findings of the cognate research on the prediction of power and electricity generation.

Esfetang and Kazemzadeh [36] developed a hybrid predictive framework combining wavelet transformation, neural network, and weight-improved particle swarm optimization (WIPSO) for precisely modeling the generation of electrical power in wind farms. Meteorological characteristics played a critical role in the entire exercise. Guo et al. [37] explored the correlation between climatic, hydrological, and socio-economic factors to forecast monthly hydroelectric generation, electricity demand, and greenhouse gas emissions. The methodological framework comprised of artificial neural network (ANN) fine-tuned through an enhanced electromagnetic field optimization algorithm (IEFO)which outclassed several benchmark tools in terms of accuracy. Şahin et al. [38] relied upon seasonal grey and machine learning-based models for estimating monthly electricity production in France, Germany, Spain, Turkey, and the UK. The methodological frameworks transpired to draw highly accurate forecasts and provided a share of renewables in total electricity generation. Research by Jiang et al. [39] successfully decoded the predictability of energy generation in electric buses using a Markov-based Gaussian Process Regression model (M-GPR), where the deployment of process-specific external variables appeared to be highly effective. Jin et al. [40] adopted deep reinforcement learning and the Markov Decision Process (MDP), closely tracking power fluctuations and predicting the overall energy consumption in buildings with high precision. The framework demonstrated statistical superiority over various competing models. Li et al. [41] designed a hybrid back propagation neural network (BPNN) optimized by the improved particle swarm (IPSO) algorithm for electricity consumption prediction during the COVID-19 pandemic regime effectively. The predictive structure utilized medical information, public opinion, policy data, and historical records of electricity consumption during the pandemic for drawing forecasts. Lu et al. [42] successfully developed a hybrid predictive framework incorporating an improved Complete Ensemble Empirical Mode Decomposition with Adaptive Noise and Support Vector Machine (SVM) for predicting daily electricity demand in the US during the COVID-19 pandemic. It was revealed that the daily infection rate largely influenced the prediction accuracy. Shi et al. [43] propounded a sliding window and dual-channel convolutional neural network for capturing temporal characteristics focused on accurately estimating both coal and electricity consumption within a specific 5-min interval during the cement calcination process. The proposed methodology outperformed a series of machine and deep learning algorithms.

The careful scrutiny of the past literature clearly elicits the scarcity of research to model clean energy generation patterns for specific resources. The past research is mostly restricted to pattern discovery of conventional coal-based energy consumption and generation pattern. However, owing to the growing need to focus on clean electricity generation, it becomes imperative to analyze the predictability of the same in practical terms, it's essential to assess the dependence structure between the energy generation and factors representing household and industrial demand to draw actionable insights. The prevailing predictive modeling literature of consumption and generation patterns sheds little light on the same. Hence, the current research's strategic alignment to address these potential gaps is well-justified. Considering the success of machine and deep learning models in forecasting future trends, the methodology outlined in this paper efficiently harnesses these models to construct a detailed predictive model. Subsequently, the underlying dependency patterns are revealed through eXplainable AI (XAI) methodologies.

## 3 Research methodology

Here, we elucidate the methodological framework designed to perform predictive modeling and subsequently explain the impact of considered explainable variables on chosen clean electricity generation series. As mentioned, the granular framework initially decomposes the underlying series into granular subseries using the EEMD method, which is subjected to the DBSCAN clustering algorithm to form the high and low-frequency series. Clustering is performed based on fuzzy entropy (FENT) and Hurst exponent (HEXP) values of the decomposed series. We explain the components used for the said restructuring at first.

### 3.1 Ensemble empirical mode decomposition (EEMD)

EEMD [44] is a method that modifies the traditional empirical mode decomposition (EMD) approach. It is applied to separate signals that are nonlinear and nonstationary into a component called intrinsic mode function (IMFs) and another component that is the residual. We briefly outline IMFs and EEMD steps.

The steps of generating IMFs:

Step 1. Find all the local minimums and maximums of the time series X(t) and use cubic spline to interpolate them to form lower envelop L(t) and upper envelop U(t).

Step 2. Compute the mean of lower and upper envelopes as equation (1):

(1) $$M\left(t\right)=\frac{L\left(t\right)+U\left(t\right)}{2}$$

Obtain a local detail as eq. (2):

(2) Z(t)=X(t)−M(t)

Step 3. Perform Steps 1 and 2 until conditions in steps 3.1 and 3.2 are satisfied:

Step 3.1. The value of M(t) limits to 0.

Step 3.2. The difference between local extrema and zero crossings is at most 1.

The first IMF, $F_{1}\left(t\right)$, is given by Z(t) provided it satisfies Steps 3.1 and 3.2. Alternatively, the maximum number of iterations can be defined instead of performing Steps 3.1and 3.2 for extracting $F_{1}\left(t\right)$. The original series is reduced by the extracted IMF to create the residual series (eq. (3)):

(3) $$R_{1}\left(t\right)=X\left(t\right)-Z\left(t\right)$$

Step 4. Repeat Steps 1 to 3 until the desired number of IMFs is extracted, and the error component has at most two local extrema or termination criteria reached. Finally, eq. (4) expresses the original time series X(t) after extracting *n* number of IMFs as:

(4) $$X\left(t\right)=\sum_{i=1}^{N}F_{i}\left(t\right)+R_{N}\left(t\right)$$

The EEMD removes the mode mixing problem of the EMD, resulting in IMF, containing signals spanning a wide band of frequency.

The steps of generating EEMD:

Step 1. Perturb the time series X(t) by adding noise components to generate the series (eq. (5)):

(5) $$X^{i}\left(t\right)=X\left(t\right)+\varepsilon \left(t\right),i\in 1\text{,}\ldots ,I$$

Where ε(t) represents independent Gaussian white noise, and *I* denotes the number of trials.

Step 2. Use EMD on individual transformed series to get the respective IMFs and residuals (eq. (6)).

(6) $$X^{i}\left(t\right)=\sum_{j=1}^{N}C_{j}^{i}+r_{N}^{i}$$

Step 3. Compute the average outcome of each trial to cancel out the effect of uncorrelated white noise

while preserving the meaningful information and getting back the original series (eq. (7)).

(7) $$X\left(t\right)=\frac{1}{I}\left(\sum_{i=1}^{I}\sum_{j=1}^{N}C_{j}^{i}+r_{N}^{i}\right)+\varepsilon_{I}$$

Where $\varepsilon_{I}=\frac{\varepsilon }{\sqrt{N}}$.

### 3.2 Density-based spatial clustering of applications with noise (DBSCAN)

It is a density-based clustering algorithm capable of automatically estimating an optimal number of clusters of any shape in a dataset [45]. It is reliant upon two important parameters, namely, *epsilon*, the distance of the vecinity around a data point, and *minPts*, referring to the number of points within the radius of epsilon to construct clusters based on the density. The parameter *epsilon* is used to classify the data points into core and noise points. The core points must exceed the *minPts*. For a data point *p* if another point *q* lies within the epsilon neighborhood, then *p* is treated as a core point, and the connection between the points is referred to as directly density reachable. The point *p* is categorized as density reachable from *q* if a succession of points $\left(p_{1},\ldots ,p_{n};\;p_{1}=p;\;p_{n}=q\right)$ exists such that $p_{i+1}$ is directly density reachable from $p_{i}$.

The algorithmic procedures of DBSCAN are enunciated below:

Step 1. Traverse the data points to discover the core points with respect to the figures of epsilon and minPts.

Step 2. Starting from any core point, mark the directly density-reachable and the density-reachable points to form a cluster.

Step 3. Perform steps 1and 2 for unvisited points.

The leftover points not assigned to any clusters are treated as noise.

### 3.3 Hurst exponent (HEXP)

This work utilizes the rescaled range (R/S) analysis-based [46] Hurst exponent [47] figure to identify the high-frequency and low-frequency counterparts of the decomposed series. The procedural steps to estimate the same are jotted down as follows.

Step 1: At first, the underlying time series (R_N_) of length N is segmented into d groups of continuous subseries of length n.

Step 2: The arithmetic mean ($M_{d}$) of each subseries T_j, d_ (j = 1, 2, …. , n) is computed.

Step 3: The cumulative deviation from the mean of the subseries is, thereafter, calculated as eq. (8):

(8) $$X_{j,d}=\sum_{i=1}^{j}\left(T_{i,d}-M_{d}\right)$$

Step 4: The range $\left(R_{d}\right)$ is determined as eq. (9):

(9) $$R_{d}=\max\left(X_{j,d}\right)-\min\left(X_{j,d}\right)$$

Step 5: The standard deviation (S_d_) of the respective subseries is calculated in eq (10).

(10) $$S_{d}=\sqrt{\left(1/n\right)\sum_{j=1}^{n}{\left(T_{j,d}-M_{d}\right)}^{2}}$$

**Step 6:** The rescaled range mean figure for the underlying sub-series is determined as eq (11):

(11) $${\left(R/S\right)}_{n}=\left(1/A\right)\sum_{d=1}^{D}\left(R_{d}/S_{d}\right)$$

The R/S statistic and Hurst coefficient (H) are asymptotically related in eq. 12

(12) $${\left(R/S\right)}_{n}=C*n^{H}$$

Wherein C is a constant

Finally, the Hurst exponent (*H*) value is estimated by applying a standard ordinary least squares regression (OLS) on eq. (13).

(13) $$\log\;{\left(R/S\right)}_{n}=\log\;C+H\;\log\;n$$

An *H* value of 0.5 implies the underlying time series perfectly follows an independent and identically distributed (iid) Gaussian Random Walk model. On the flip side, if its value is greater than 0.5, a persistent presence trend characterized by long-memory dependence is concluded. Time series characterized by long-memory dependence ideally suggests the dominance of trend component, inferring low-frequency traits. The presence of high-frequency components is inferred if the estimated figure of H is less than 0.5, which suggests strong dominance of an anti-persistent pattern. We determine the values of H of respective decomposed series of selected energy generation indicators.

### 3.4 Fuzzy entropy

Entropy is an effective reflector of the extent of disorder in thermodynamic systems. It has been successfully used to gauge the degree of complexity and volatility of time series data [48]. In this work, we rely upon the fuzzy entropy measure used by Ref. [49] for forecasting Carbon futures for clustering decomposed components of respective energy generation series into sub-series. The procedural steps are outlined below.

Step 1: For a given time series, x(t) with embedding dimension m, the m dimensional vector representation is expressed as eq. (14):

(14) $$X_{i}^{m}=\left[x\left(i\right),x\left(i+1\right),\ldots ,x\left(i+m-1\right)\right]$$

Where 1≤i≤T−m+1.

Step 2: Eq. (15) shows the distance between two vectors, X(i), X(j):

(15) dijm=d[Xim,Xjm]={|[x(i+k)−x(j)]−[x(i+k)−x(j)]|}k=0,1,…,m−1max

for i,j=1,2,…,T−m,i≠j.

Step 3: The similarity $\left(D_{ij}^{m}\right)$ between $X_{i}^{m}$ and $X_{j}^{m}$ is determined by estimating the fuzzy membership function as eq. (16):

(16) $$D_{m}^{ij}=\mu \left(d_{ij}^{m},n.r\right)=\exp\left(-{\left(d_{ij}^{m}/r\right)}^{n}\right)$$

where *n* is a parameter, and *r* represents a tolerance parameter.

Step 4: The following functions are calculated on top of the fuzzy similarity score (eqs (17), (18))):

(17) $$\varphi^{m}\left(n,r\right)=\frac{1}{T-m+1}\sum_{i=1}^{T-m+1}\left(\frac{1}{T-m}\sum_{j=1;i\neq j}^{T-m+1}D_{ij}^{m}\right)$$

(18) $$\varphi^{m+1}\left(n,r\right)=\frac{1}{T-m}\sum_{i=1}^{T-m}\left(\frac{1}{T-m-1}\sum_{j=1;i\neq j}^{T-m}D_{ij}^{m+1}\right)$$

Step 5: Finally, Eq. (19) computes the fuzzy entropy of the time series, x(t):

(19) $$FuzzyEntropy\left(m,n,r,T\right)=\ln\;\varphi^{m}\left(n,r\right)-\ln\;\varphi^{m+1}\left(n,r\right)$$

The value of r, denoting the fuzzy function extent of the border, is set as $0.25\sigma_{SD}$, where $\sigma_{SD}$ denotes the time series standard deviation, the embedding dimension, m is set to be 3, and the value of n assigned to 2 in this work. The figures of fuzzy entropy for respective decomposed subseries alongside the trend component are estimated. Higher entropy values indicate high variability and thereby show high-frequency components.

**Fig. 1:**
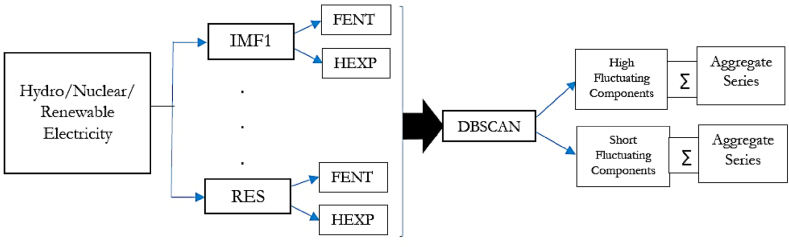
Restructuring the hydro/nuclear/renewable electricity generation.

Fig. 1 depicts the integrated process to generate the aggregate series from hydro/nuclear/renewable electricity generation series.

The ASO-based predictive structure is applied to the reconstructed aggregate series of electricity generation from respective clean resources. We run the predictive framework on both aggregate high and low component series separately for individual series. The estimated forecasts on both counterparts are added to obtain the final predictions. The ASO algorithm is used in an optimization setup to combine predictions obtained from the individual ensemble models, RF, Bagging, and GB. Thus, the approach can alternatively be classified as EoE framework. The descriptions of the constituent components are elaborated below.

### 3.5 Atom search optimization (ASO)

Propounded by Zhao et al. [50], ASO is a population-based metaheuristic search algorithm mimicking molecular dynamics. It is inspired by the natural atomic motion, subjected to interaction forces and geometric constraints. Each atom represents a potential solution in search space and traverses toward the target via acceleration. Atoms interact with each other in the feasible domain to obtain the best solution. The motion of an atom is described using equations (20), (21)).

(20) $$v_{i}^{d}\left(t+1\right)={rand}_{i}^{d}v_{i}^{d}\left(t\right)+a_{i}^{d}\left(t\right)$$

(21) $$x_{i}^{d}\left(t+1\right)=x_{i}^{d}\left(t\right)+v_{i}^{d}\left(t+1\right)$$

Where *i* denotes an atom in the population, d=1,…,D; D accounts for the number of decision variables, $v_{i}^{d}$, and $x_{i}^{d}$ are the speed and location of the *i*th atom, *t* stands for the iteration, rand is random number in the range [0, 1], and $a_{i}^{d}$ is the acceleration component.

Considering the interaction force and geometric constraints, the acceleration of atoms is estimated using the principles of Newtonian mechanics as expressed in equation (22).

(22) $$a_{i}^{d}\left(t\right)=\frac{F_{i}^{d}\left(t\right)}{m_{i}^{d}\left(t\right)}+\frac{G_{i}^{d}\left(t\right)}{m_{i}^{d}\left(t\right)}=-\alpha {\left(1-\frac{t-1}{T}\right)}^{3}e^{-\frac{20t}{T}}\sum_{j\in K_{best}}\frac{{rand}_{j}\left[2\times {\left(h_{ij}\left(t\right)\right)}^{13}-{\left(h_{ij}\right)}^{7}\right]}{m_{i}\left(t\right)}\frac{\left(x_{j}^{d}\left(t\right)-x_{i}^{d}\left(t\right)\right)}{{\left\|x_{i}\left(t\right),x_{j}\left(t\right)\right\|}_{2}}+\beta e^{-\frac{-20t}{T}}\frac{x_{best}^{d}\left(t\right)-x_{i}^{d}\left(t\right)}{m_{i}\left(t\right)}$$

The interaction fore and the geometric constraint between the atoms are represented by $F_{i}^{d}$ and $G_{i}^{d}$, α represents the depth weight, β stands for multiplier weight, $h_{ij}$ is an adaptive ratio of Euclidean distance between atom i and j, to the distance at which the inter-particle potential is 0, T is the maximum number of repetitions. The interaction force, geometric constant, and mass of the respective particles are determined using equations (23), (24), (25), (26).

(23) $$F_{i}^{d}\left(t\right)=\sum_{j\in K_{best}}{rand}_{j}F_{ij}^{d}\left(t\right)=\sum_{j\in K_{best}}-{rand}_{j}*\alpha {\left(1-\frac{t-1}{T}\right)}^{3}e^{-\frac{20t}{T}}\left[2{\left(h_{ij}\left(t\right)\right)}^{13}-{\left(h_{ij}\left(t\right)\right)}^{7}\right]$$

(24) $$G_{i}^{d}\left(t\right)=\beta e^{-\frac{-20t}{T}}\left(x_{i}^{d}\left(t\right)-x_{best}^{d}\left(t\right)\right)$$

(25) $$M_{i}^{d}\left(t\right)=e^{-\frac{{Fit}_{i}\left(t\right)-{Fit}_{best}\left(t\right)}{{Fit}_{worst}\left(t\right)-{Fit}_{best}\left(t\right)}}$$

(26) $$m_{i}^{d}\left(t\right)=\frac{M_{i}\left(t\right)}{\sum_{j=1}^{N}M_{j}\left(t\right)}$$

Here, $F_{ij}^{d}\left(t\right)$ is the interaction force between atoms *i* and *j*, and $K_{best}$ denotes the collection of best K atoms as per the fitness. Thus, individual atoms are engaged in transfusing information with *K* neighbors. The best and the worst fitness values at iteration *t* are denoted by ${Fit}_{best}\left(t\right)$ and ${Fit}_{worst}\left(t\right)$. The neighborhood size is controlled over the iterations as follows (eq (27)):

(27) $$K\left(t\right)=N-\left(N-2\right)\times \sqrt{\frac{t}{T}}$$

*N* denotes the population size.

The methodological framework to perform predictive modeling attempts to utilize the capacity of the ASO algorithm, which has been highly regarded for resolving complex NP-Complete optimization problems [51,52], to intelligently traverse the search space for combing three ensemble learning algorithms. The rationale for the selection of the ASO algorithm also covers the endeavor to explicate its theoretical potential in building a sophisticated granular time series forecasting methodology. The faster convergence and ability to penetrate the farthest solution space by thwarting local optimal make it ideal for complex optimization problems. The ASO-based EoE structure alternatively can be regarded to be an ensemble of ensembles owing to the flow of operations. The novelty of the predictive frame lies in adopting ASO in building the EoE structure for predicting a pattern of paramount significance in the context of sustainability. Here, we enunciate a brief overview of the three ensemble models.

### 3.6 Random forest (RF)

Breiman [53] developed RF, a typical ensemble machine learning algorithm predominantly used for predictive modeling. It uses a set of base learning algorithms in parallel for making the initial predictions. Generally, the orthodox decision tree is used as base learners. In this work, the classical regression tree (CART) has been used for that role. Each base learner is constructed based on a bootstrapped data segment of the entire training data samples. At each node of the selected tree, the best feature for the split is determined based on a randomly chosen subset of features. The final prediction is obtained by averaging the individual base learners estimates. These algorithms have been successfully applied in the predictive modeling of financial time series [54,55].

### 3.7 Bagging

Originated by Breiman [56], bagging is another ensemble machine learning algorithm wherein a series of CART is deployed as the base learners in parallel. Similar to RF, the base learners are built on bootstrapped samples. However, it does not select features in underlying base learners on a random subset of features. Unlike RF, all features are evaluated to identify the most suitable one, thereby striving to reduce the variance of unstable learning in CART. The final predicted value is the average of all the individual learners. Bagging has been successfully used for complex pattern mining [57].

### 3.8 Gradient boosting (GB)

Propounded by Schapire and Singer [58], Boosting is also an ensemble machine-learning technique that applies a series of base learning algorithms forward-stage-wise to produce the final predictions. The GB is a variant of the traditional boosting algorithm that uses gradient-driven error rate for the identification of training samples. As individual learners, classical regression trees are trained in each stage sequentially in a forward direction. The sequential ensemble approach assists in reducing the inaccuracy of the prediction. Akin to RF and bagging, GB has been found to be highly effective in mining complex patterns [59,60].

The entire simulation has been performed using Python programming language, wherein the *‘GridSearchCV’* utility of the *‘sklearn’* library is used for parameter tuning of respective methods. The *‘mealpy’* library is used for implementing the ASO algorithm.

### 3.9 Optimization framework for drawing final predictions

As discussed, the ASO framework is utilized to combine the energy consumption forecasts produced by RF, Bagging, and GB to fetch the final prediction. The ASO-based structure computes the weighted average of individual predictions for drawing the final outcome. The mathematical formulation is narrated in equation (28).

(28) $$Y_{i}^{Fin}=w_{1}Y_{i}^{RF}+w_{2}Y_{i}^{Bagging}+w_{3}Y_{i}^{GB}$$

Here, $Y_{i}^{RF}$, $Y_{i}^{Bagging}$, and $Y_{i}^{GB}$ denote predictions on ith sample by RF, Bagging, and GB methods; $Y_{i}^{Fin}$ represents the final predicted figure by the ASOE model; and $w_{1}$, $w_{2}$, $w_{3}$ are respective weighted contributions of the respective ensemble models. The weighted average framework follows the following constraint (eq. (29)).

(29) $$w_{1}+w_{2}+w_{3}=1$$

For finding the optimal weights, we propose the following optimization problem (eq. (30)):

(30) $$\min:z=\sum_{i=1}^{N}{\left(Y_{i}^{Actual}-Y_{i}^{Fin}\right)}^{2}$$

The objective function mentioned in equation (6) can be represented by following an equivalent optimization framework (eq. (31), (32), (33), (34), (35), (36), (37), (38)).

(31) $$\min:z=\sum_{i=1}^{N}{\left\{Y_{i}^{Actual}-\left(w_{1}Y_{i}^{RF}+w_{2}Y_{i}^{Bagging}+w_{3}Y_{i}^{GB}\right)\right\}}^{2}$$

subject to

$$\sum_{i=1}^{N}{\left\{Y_{i}^{Actual}-\left(w_{1}Y_{i}^{RF}+w_{2}Y_{i}^{Bagging}+w_{3}Y_{i}^{GB}\right)\right\}}^{2}$$

(32) $$\leq \sum_{i=1}^{N}{\left(Y_{i}^{Actual}-Y_{i}^{RF}\right)}^{2}$$

$$\sum_{i=1}^{N}{\left\{Y_{i}^{Actual}-\left(w_{1}Y_{i}^{RF}+w_{2}Y_{i}^{Bagging}+w_{3}Y_{i}^{GB}\right)\right\}}^{2}$$

(33) $$\leq \sum_{i=1}^{N}{\left(Y_{i}^{Actual}-Y_{i}^{Bagging}\right)}^{2}$$

$$\sum_{i=1}^{N}{\left\{Y_{i}^{Actual}-\left(w_{1}Y_{i}^{RF}+w_{2}Y_{i}^{Bagging}+w_{3}Y_{i}^{GB}\right)\right\}}^{2}$$

(34) $$\leq \sum_{i=1}^{N}{\left(Y_{i}^{Actual}-Y_{i}^{GB}\right)}^{2}$$

(35) $$w_{1}+w_{2}+w_{3}=1$$

(36) $$0\leq w_{1}\leq 1$$

(37) $$0\leq w_{2}\leq 1$$

(38) $$0\leq w_{3}\leq 1$$

The ASO algorithm is deployed to iteratively fetch the near-optimal values of the respective weights to augment the accuracy of the final predictions. To assess the predictive performance, the present research utilizes four metrics as defined next.

### 3.10 Performance indicators

The following criteria are used to measure how well the proposed XAI framework performs. Let $\left(Y_{t}\right)$ denotes the observed series and $\left({\hat{Y}}_{t}\right)$ denote the estimated series. Then the four measures, Nash-Sutcliffe Efficiency (NSE), Index of Agreement (IA), Theil Index (TI), and Directional Predictive Accuracy (DA) are defined as follows (eq. (39), (40), (41), (42))):

(39) $$NSE=1-\frac{\sum_{t=1}^{N}{\left\{Y_{t}-{\hat{Y}}_{t}\right\}}^{2}}{\sum_{t=1}^{N}{\left\{Y_{t}-\bar{Y}\right\}}^{2}}$$

(40) $$IA=1-\frac{\sum_{t=1}^{N}{\left(Y_{t}-{\hat{Y}}_{t}\right)}^{2}}{\sum_{t=1}^{N}{\left\{\left|{\hat{Y}}_{t}-\bar{Y}\right|+|Y_{t}-\bar{Y}|\right\}}^{2}}$$

(41) $$TI=\frac{{\left[\frac{1}{N}\sum_{t=1}^{N}{\left(Y_{t}-{\hat{Y}}_{t}\right)}^{2}\right]}^{1/2}}{{\left[\frac{1}{N}\sum_{t=1}^{N}{\left({\hat{Y}}_{t}\right)}^{2}\right]}^{1/2}+{\left[\frac{1}{N}\sum_{t=1}^{N}{\left(Y_{t}\right)}^{2}\right]}^{1/2}}$$

(42) $$DA=\frac{\sum_{t=1}^{N}\left\{ \begin{array}{c} 1,if\;\left(Y_{t+1}-Y_{t}\right)\left({\hat{Y}}_{t+1}-Y_{t}\right)\geq 0\\ 0,Otherwise\; \end{array}\right.}{N}$$

A predictive modeling approach will be highly efficient if the *NSE*, *IA*, *and DA* values approach to one and the *TI* value approach to zero. The Diebold-Mariano (DM) test for equal predictability analysis is used in this work to statistically ascertain the competing models' relative efficiency. The DM test can evaluate the accuracy differences between multiple forecasting models using mean-squared residual. We compare the models using mean square prediction error (MSPE) as the loss function.

The integrated approach is sufficient to gauge the extent of predictability of the underlying, but it reduces model interpretability. To overcome this limitation, the current work uses dedicated XAI tools, explained below.

### 3.11 Explainable artificial intelligence (XAI)

To understand how the selected explanatory features affect the respective electricity generation series, the current work invokes several XAI models outlined below to accomplish the task.

#### 3.11.1 Permutation feature importance

Breiman [53] originally developed orthodox permutation feature importance to understand the effect of the explanatory features in a random forest model. Fisher et al. [61] later extended it to use it as a model agnostic tool. In the updated scheme, the importance of any feature is calculated by randomly changing its original values and measuring the impact on the overall predictive accuracy of the model. A higher error means a higher importance of the feature.

#### 3.11.2 Accumulation local effect plot

The accumulation local effect (ALE) plot was initially introduced by Apley and Zhu [62], which measures the average impact of chosen independent variables on the predictions of developed machine learning models by exposing the black box operations. ALE plots are quicker to build and give correct interpretation in the case of correlated features, unlike the PDP plots.

The local-dependence (LD) profile for a model f() and predictor $X^{j}$ was later developed by Apley and Zhu [63] as (eq. (43)):

(43) $$g_{LD}^{f,j}\left(z\right)=E_{{\underline{X}}^{-j}|X^{j}=z}\left\{f\left({\underline{X}}^{j|=z}\right)\right\}$$

Basically, it is the expected value of the model predictions over the conditional distribution of ${\underline{X}}^{-j}$ given $X^{j}=z$.

The LD profile is calculated as follows (eq. (44)):

(44) $${\hat{g}}_{LD}^{j}\left(z\right)=\frac{1}{\left|N_{j}\right|}\sum_{k\in N_{j}}f\left({\underline{x}}_{k}^{j|=z}\right)$$

Where $N_{j}$ represents the set of observations utilized for determining the conditional distribution of ${\underline{X}}^{-j}|X^{j}=z$, with the values of $X^{j}$ close to z.

A smooth estimator for the LD profile can be written as (eq. (45)):

(45) $${\hat{g}}_{LD}^{j}\left(z\right)=\frac{1}{\sum_{k}w_{k}\left(z\right)}\sum_{i=1}^{n}w_{i}\left(z\right)f\left(x_{-i}^{j|=z}\right)$$

The weight components $w_{i}\left(z\right)$ indicates the distance between z and $x_{i}^{j}$.

The Accumulated-local (AL) profile for the model f() and predictor $X^{j}$ is estimated as (eq. (46)):

(46) $$g_{AL}^{j}\left(z\right)=\int_{z_{0}}^{z}\left[E_{{\underline{X}}^{-j}|X^{j}=z}\left\{q^{j}\left({\underline{X}}^{j|=z}\right)\right\}\right]dv+c$$

Where $q_{j}\left(u\right)={\left\{\frac{\partial f\left(\underline{x}\right)}{{\partial x}^{j}}\right\}}_{\underline{x}=\underline{u}}$, $z_{0}$ shows a value near the lower bound of the distribution $X^{j}$, and c is the constant.

The local variation of the model due to $X^{j}$ is measured by $q_{j}\left({\underline{x}}^{j|=v}\right)$. The amount of changes is averaged to calculate the accumulation of local effects. The mentioned formulation is very effective in exploring contributions for correlated feature.

#### 3.11.3 Local interpretable model-agnostic explanations

Local Interpretable Model-Agnostic Explanations (LIME) is an explainable AI model to understand machine learning models locally, developed by Ribiero et al. [64]. LIME generates a novel dataset based on a learned model by modifying the input variables' figures and getting the target variable's predictions. It then attempts to map the relationship between the target and input variables on the new dataset using more interpretable machine learning models, viz., decision trees, LASSO, etc. It is mostly used to assess the local influence structure of the explanatory feature set.

## 4 Data description & statistical properties

**Fig. 2:**
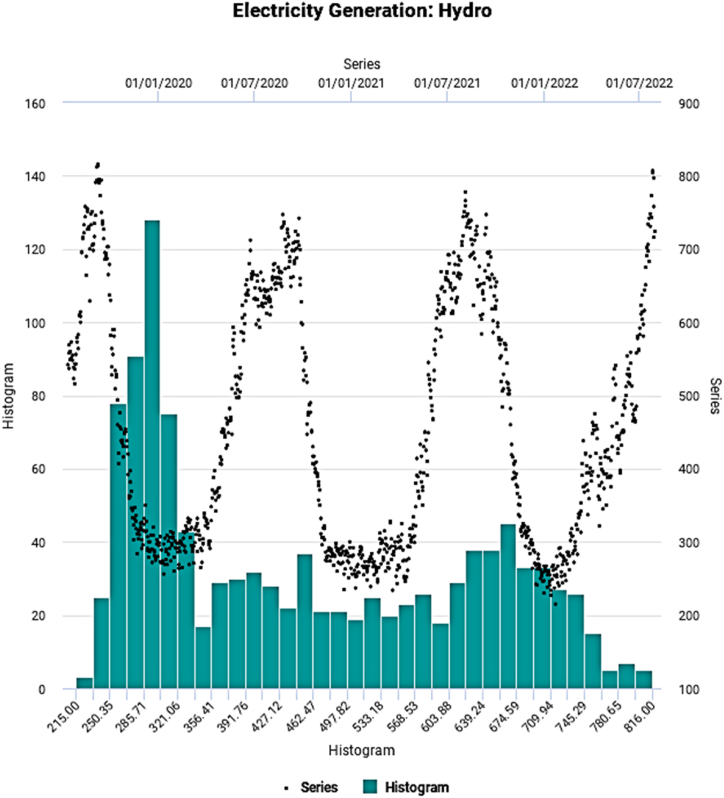
Evolutionary pattern of hydro electricity generation.

**Fig. 3:**
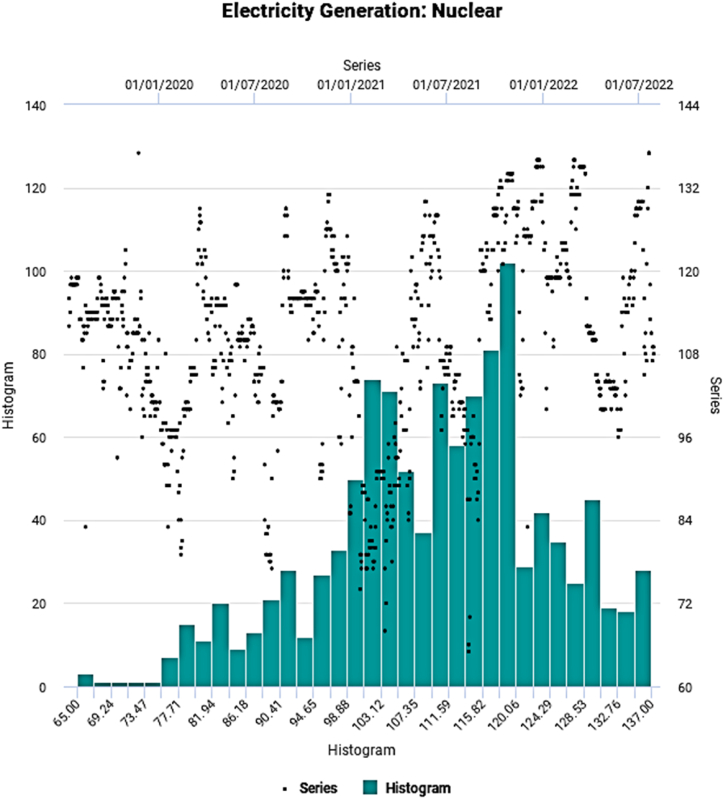
Evolutionary pattern of nuclear electricity generation.

**Fig. 4:**
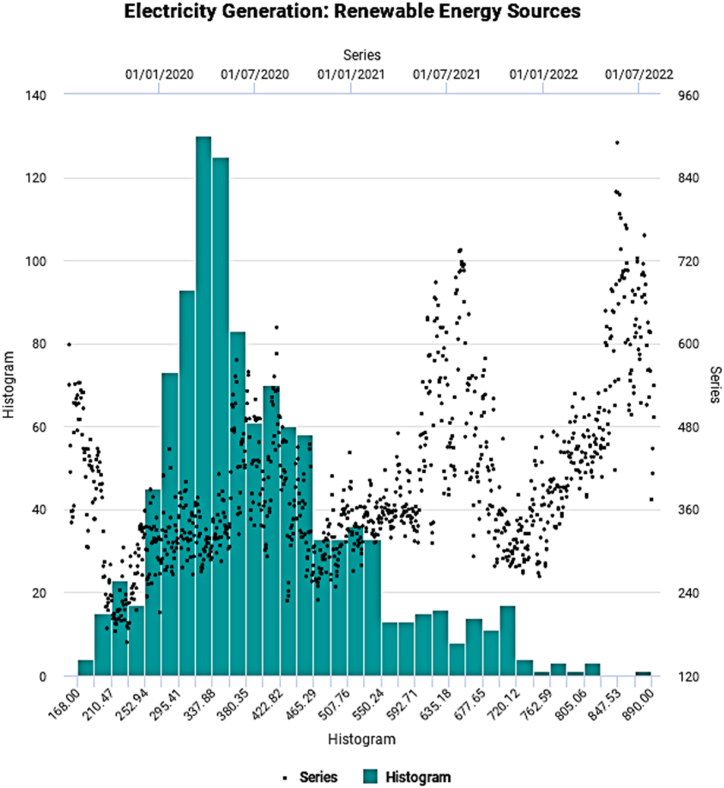
Evolutionary pattern of renewable electricity generation.

The CEIC global database [65] is used for collating data of variables for conducting the experiments. The samples of daily hydro, nuclear, and renewable energy generation in a million units (MU) of India from July 15, 2019, to June 30, 2022, are compiled to test the predictability. Renewable electricity is extracted from sources comprising solar, biomass, etc. The sample duration of the study duly covers the COVID-19 pandemic time horizon, which underscores the contribution of the study. The following exhibits, Fig. 2, Fig. 3, Fig. 4, provide the visual depiction of the original series and respective histograms.

The evolutionary temporal pattern appears to exhibit cyclic and steep nonlinear movements over time. Table 2 shows the main statistical properties of the underlying energy generation series.

**Table 2:** Key statistical characteristics.

Series	Hydro	Nuclear	Renewable
Minimum	241.0	66.0	184.0
Maximum	816.0	136.0	818.0
Mean	451.4	109.8	394.6
Median	409.5	111.0	363.5
Std. Dev.	163.67	13.68	114.91
Skewness	0.438	−0.388	1.012
Kurtosis	−1.278	−0.244	0.919
SW Test	0.890^a^	0.983^a^	0.933^a^
AD Test	29.483^a^	2.918^a^	14.808^a^
ADF Test	−0.643#	−0.602#	−1.498#
Terasvirta's NN Test	505.28^a^	484.13^a^	527.91^a^
Hurst Exponent	0.739^a^	0.774^a^	0.793^a^

a Significant at 1 % Level of Significance, #Not Significant, SW Test: Shapiro-Wilk Test, AD Test: Anderson-Darling Test, ADF Test: Augmented Dickey-Fuller Test, Terasvirta's NN Test: Terasvirta's Neural Network Test.

The measure of dispersion, Std. Dev. Indicates a relatively high degree of variation for hydro and renewable electricity generation than nuclear-based one implying considerably higher demand fluctuation. All underlying time series emerge to be nonparametric, as manifested by the SW and AD test statistics. On the other hand, insignificant ADF test statistic figures suggest nonstationary behavior. Finally, as apparent from the visual inspection, the presence of nonlinearity conforms with the outcome of Terasvirta's NN Test.

The daily Google search trends in India on six topics, Government Subsidy (Subsidy), Udemy, Zoom, Amazon, Coursera, and Unemployment, are hosted in the CEIC database, which is collated to reflect the sentiment of the household and gauges the engagement with different activities striving on electricity consumption. The search topics are selected considering the study's timeline and the Indian context. We have carefully attempted to incorporate the degree of engagement in online learning and work from home, buying behavior, effects of apprehension, and reliance on government support of common people to represent the state of household in influencing electricity intake through the chosen search indicators. The utilization of the six indicators is meaningful in the COVID-19 pandemic scenario. To incorporate the demand side effects of industrial production, daily closing prices of sectoral indices, namely, Power, Energy, Automobile (Auto), Fast Moving Consumer Goods (FMCG), Capital Goods (Cap_Goods), Telecommunication (Telecom), Healthcare, and Consumer Durable (Con_Durable) have been chosen as explanatory features. The data on sectoral market prices are collected from the official portal of Investing.com [66]. Deploying sectoral indices account for the overall industrial growth and appetite for energy intake in India.

## 5 Results & analyses

In this section, we elucidate the detailed outcomes of predictions and interpretation.

### 5.1 Predictive modeling

**Fig. 5:**
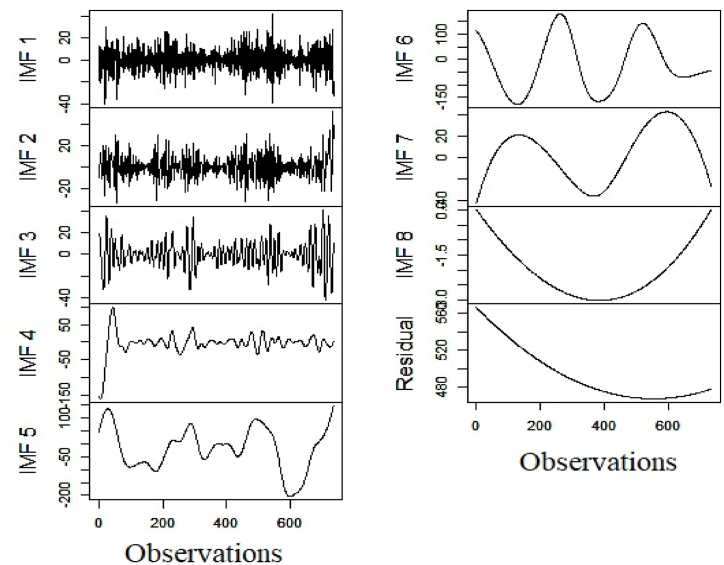
Decomposition of hydro electricity generation series.

**Fig. 6:**
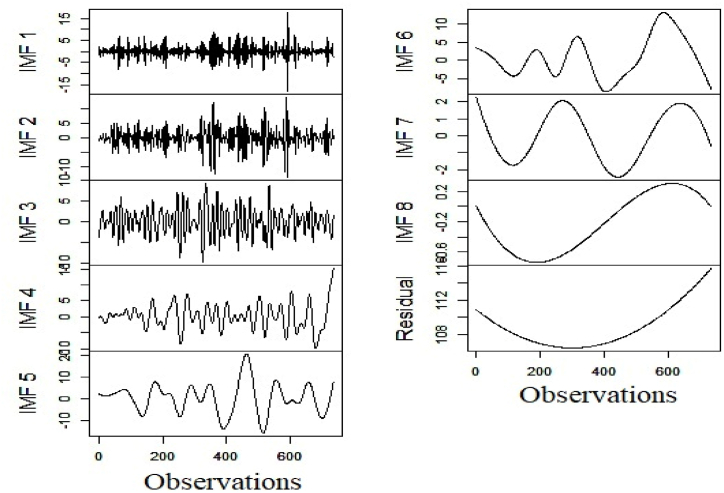
Decomposition of nuclear electricity generation series.

**Fig. 7:**
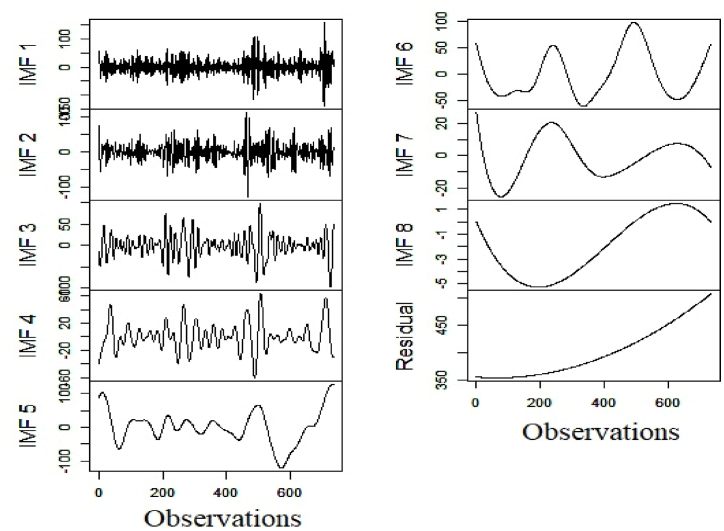
Decomposition of renewable electricity generation series.

As discussed, the first stage of the predictive modeling commences with the decomposing of the original daily electricity generation series for identifying the high and low-frequency counterparts subsequently through the clustering framework. Fig. 5, Fig. 6, Fig. 7 represent the outcome of the decomposition process and the resultant IMFs of respective series through the EEMD methodology.

After completion of the decomposition of the respective series, we estimate the values of HEXP and FENT of the underlying subseries. The estimated figures are treated as features for DBSCAN clustering. Table 3 reports the results of the granular time series clustering (Low/High Frequency).

**Table 3:** Outcome of granular time series clustering.

Series	Fuzzy Entropy	Hurst Exponent	Clustering Outcome
Hydro Electricity			
IMF1	3.590	0.358	High Freq.
IMF2	2.923	0.484	High Freq.
IMF3	2.312	0.487	High Freq.
IMF4	1.649	0.708	High Freq.
IMF5	1.810	0.780	High Freq.
IMF6	1.699	0.758	High Freq.
IMF7	0.344	0.838	Low Freq.
IMF8	0.001	0.872	Low Freq.
Residual	0.045	0.869	Low Freq.
**Nuclear Electricity**			
IMF1	1.835	0.400	High Freq.
IMF2	1.502	0.481	High Freq.
IMF3	1.030	0.486	High Freq.
IMF4	0.475	0.658	High Freq.
IMF5	0.343	0.712	High Freq.
IMF6	0.049	0.822	Low Freq.
IMF7	0.002	0.809	Low Freq.
IMF8	0.000	0.880	Low Freq.
Residual	0.001	0.863	Low Freq.
**Renewable Electricity**			
IMF1	4.314	0.389	High Freq.
IMF2	3.671	0.416	High Freq.
IMF3	2.867	0.563	High Freq.
IMF4	1.920	0.615	High Freq.
IMF5	1.493	0.781	Low Freq.
IMF6	1.054	0.805	Low Freq.
IMF7	0.184	0.796	Low Freq.
IMF8	0.001	0.880	Low Freq.
Residual	0.051	0.871	Low Freq.

After the successful segregation of decomposed components into high and low-frequency buckets, we aggregate to form composite high and low-frequency categories of select clean electricity generation series as visually shown in Fig. 1, Fig. 2, Fig. 3. The ASO-based predictive setup is applied for drawing predictions. The whole samples of the respective series have been split into two different data partitions, 70%–30 %, and 85%–15 %, for training and testing the model, respectively. The partition has been forward-looking, which has been reported as an ideal setup for analyzing the predictability of the financial time series [55]. Consequently, the test phase considerably covers the COVID-19 horizon, enabling the volatile phase to evaluate the predictability. Table 4, Table 5 report the outcome of predictive exercises in terms of performance metrics.

**Table 4:** Outcome of predictive exercises on 70%–30 % setup.

Electricity	NSE	TI	IA	DA
Training Segment				
Hydro	0.98536	0.03278	0.98841	0.99222
Nuclear	0.98102	0.03436	0.98353	0.99222
Renewable	0.97229	0.03681	0.97416	0.98833
		**Test Segment**		
Hydro	0.97424	0.03584	0.98335	0.98636
Nuclear	0.96898	0.03723	0.97584	0.98182
Renewable	0.96137	0.03915	0.96790	0.97727

**Table 5:** Outcome of predictive exercises on 85%–15 % setup.

Electricity	NSE	TI	IA	DA
Training Segment				
Hydro	0.99108	0.03185	0.99295	0.99519
Nuclear	0.98472	0.03328	0.98560	0.99359
Renewable	0.97755	0.03572	0.97844	0.98878
		**Test Segment**		
Hydro	0.98623	0.03302	0.98947	0.99091
Nuclear	0.97664	0.03618	0.97802	0.98182
Renewable	0.97128	0.03834	0.97256	0.97272

The figures of the NSE and IA for individual electricity generation series have emerged to be on the higher side, greater than 0.97 on training and 0.96 on the test segment. In addition, the TI values have turned out to be reasonably low in both segments. Therefore, inference can be drawn that the proposed ASO-based granular predictive framework has successfully decoded the inherent pattern of hydro, nuclear, and renewable electricity generation and estimated accurate predictions. The computed DA is close to 1 in both training and test segments, which exemplifies the predictive model's capacity to forecast the trend movements precisely. The effectiveness in estimating accurate directional changes is of paramount practical relevance as quick and precise anticipation of futuristic trends is useful for drawing roadmaps to control clean electricity generation. Among the three electricity generation series, hydro electricity generation is more predictable, followed by nuclear and renewable. We next assess the predictability in an 85%–15 % setup.

Similar results to that of 70%–30 % data split have prevailed for this setup as well. High NSE, IA, and DA values can be observed in both training and test segments, while considerably low TI values are linked with train and test sections. Marginal improvement in overall accuracy is apparent, too, as manifested by the respective performance metrics. An increase in more training samples largely accounts for the phenomenon. The propounded ASO-driven granular forecasting methodology can precisely predict both absolute figures and trends. Hydro electricity generation in the said setup has appeared to be relatively more predictable, too, followed by nuclear and renewable counterparts. The quality of prediction on both configurations truly rationalizes the utility and capability of the predictive structure in modeling clean electricity generation in India. As the samples of the study suitably cover the COVID-19 pandemic regime and the ongoing Russia-Ukraine military conflict, the ability to derive predictions of supreme accuracy in turbulent times is truly established.

Although the result of the predictive exercises confirms the utility of the methodological framework in precisely estimating the clean electricity trend, it is essential to conduct a comparative performance evaluation against several benchmark models to explain the usage holistically.

### 5.2 Comparative performance evaluation

We consider the individual ensemble machine learning models, RF, Bagging, and GB, as competing models to gauge the advantage of the ASO in combining the forecasts from individual models. The respective electricity generation series are decomposed using the EEMD procedure to facilitate the forecasting process of the competing models. The RF, Bagging, and GB are applied to draw forecasts on the decomposed series, which are aggregated to produce the final output. The DBSCAN-based clustering framework is not used to identify the high and low-frequency counterparts. The said design effectively captures the contribution of the clustering component on the overall performance of the granular predictive methodology. Lastly, support vector regression (SVR) has been used as a standalone machine learning-based competing model.

As stated earlier, the DM statistical test is invoked to compare the efficacy of the proposed predictive structure over the competing models. The test involves paired comparisons, so the order of the pair members is important for the final interpretation. The competing models are numbered in parentheses to show the order. If the test statistic is positive and significant, the model with number 2 in parentheses is considered to have statistically better forecasts than the model with number 1. Conversely, if the test statistic is negative and significant, the opposite is true, i.e., the model with number 1 in parentheses is statistically better than the model with number 2. Finally, if the test statistic is not significant, it is assumed that there is no significant difference between the models in prediction accuracy. Table 6, Table 7 outline the outcome.

**Table 6:** Result of DM test for 70%–30 % setup.

Models	SVR (1)	EEMD-RF (1)	EEMD-Bagging (1)	EEMD-GB (1)
			Hydro Electricity	
SVR (2)	–			
EEMD-RF (2)	4.860***	–		
EEMD-Bagging (2)	4.872***	0.225^a^	–	
EEMD-GB (2)	4.898***	0.237^a^	0.211#	–
Proposed (2)	7.425***	6.868***	6.914***	6.843***
			Nuclear Electricity	
SVR (2)	–			
EEMD-RF (2)	4.746***	–		
EEMD-Bagging (2)	4.805***	0.224^a^	–	
EEMD-GB (2)	4.823***	0.209^a^	0.238#	–
Proposed (2)	7.514***	6.851***	6.887***	6.864***
			Renewable Electricity	
SVR (2)	–			
EEMD-RF (2)	4.779***	–		
EEMD-Bagging (2)	4.841***	0.242^a^	–	–
EEMD-GB (2)	4.802***	0.229^a^	0.204#	–
Proposed (2)	7.592***	6.889***	6.924***	6.916***

a Not Significant, ***Significant at 1 % Level of Significance.

**Table 7:** Result of DM test for 70%–30 % setup.

Models	SVR (1)	EEMD-RF (1)	EEMD-Bagging (1)	EEMD-GB (1)
			Hydro Electricity	
SVR (2)	–			
EEMD-RF (2)	4.815***	–		
EEMD-Bagging (2)	4.794***	0.241#	–	
EEMD-GB (2)	4.826***	0.230#	0.239#	–
Proposed (2)	7.387***	6.844***	6.914***	6.825***
			Nuclear Electricity	
SVR (2)	–			
EEMD-RF (2)	4.769***	–		
EEMD-Bagging (2)	4.784***	0.232#	–	
EEMD-GB (2)	4.816***	0.215#	0.211#	–
Proposed (2)	7.596***	6.904***	6.937***	6.919***
			Renewable Electricity	
SVR (2)	–			
EEMD-RF (2)	4.656***	–		
EEMD-Bagging (2)	4.671***	0.213#	–	–
EEMD-GB (2)	4.740***	0.227#	0.233#	–
Proposed (2)	7.417***	6.814***	6.835***	6.852***

#Not Significant, ***Significant at 1 % Level of Significance.

The outcome of the pairwise DM tests provides an outright indication of the superiority of the proposed predictive model over all competing models for hydro, nuclear, and renewable electricity generation prediction processes. Therefore, the utility of the ASO-driven optimization approach on top of clustered granular series for forecasting future figures can be concluded to augment the quality of predictions significantly. It should be noted that merely decomposing the series by EEMD procedure to facilitate the prediction task may be unable to explain abrupt random variations. However, the three competing models, EEMD-RF, EEMD-Bagging, and EEMD-GB, have emerged to be superior to the standalone SVR model, which clearly emphasizes the advantage of EEMD methodology for modeling complex time series at a granular level.

A similar outcome is apparent in the 85%–15 % setup, exemplifying the statistical superiority of the proposed ASO-driven EoE framework over the competing ones. Incorporating EEMD significantly improved the accuracy, as manifested by the relatively superior form of EEMD-RF, EEMD-Bagging, and EEMD-GB over standalone SVR.

Thus, the overall comparative evaluation by DM test across both setups espouses the efficacy of the proposed granular predictive architecture. The deployment of a clustering-based decomposition process and subsequent systematic combination of the individual ensemble learning models through metaheuristic search algorithms enable the methodology to outshine the competing frameworks. The robustness of the developed forecasting architecture to predict clean electricity generation in India during the turmoil regime is proven. We next proceed to modeling by XAI methodologies for interpreting the role of the explanatory features.

### 5.3 Model interpretation through XAI

We aim to derive feature interpretation globally by permutation feature evaluation, ALE plots, and locally by LIME plots. The findings are discussed sequentially.

#### 5.3.1 Outcome of permutation feature evaluation

**Fig. 8:**
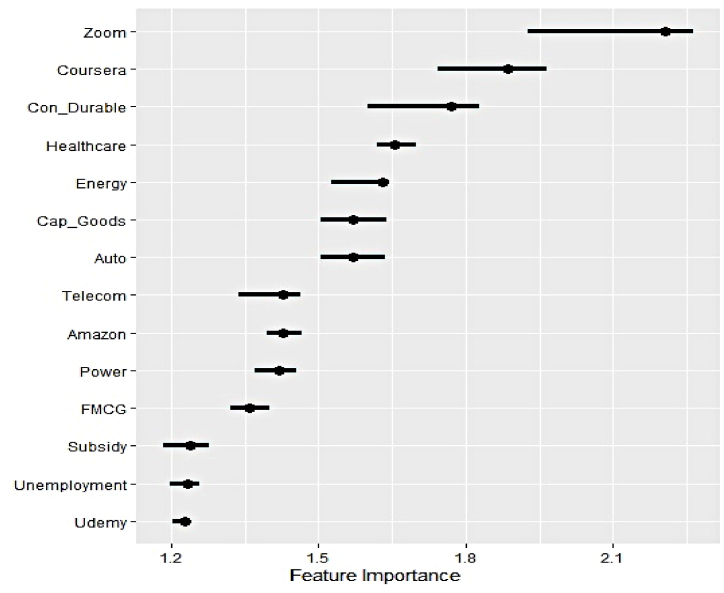
Global feature ranking for hydro electricity generation.

**Fig. 9:**
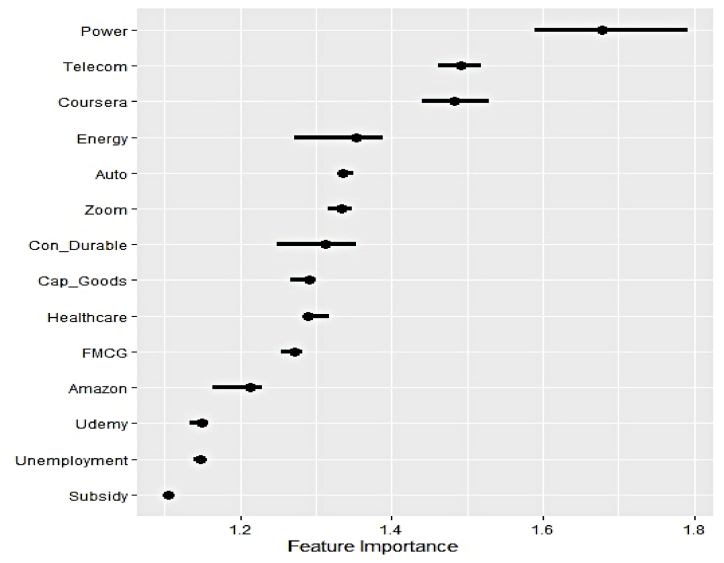
Global feature ranking for nuclear electricity generation.

**Fig. 10:**
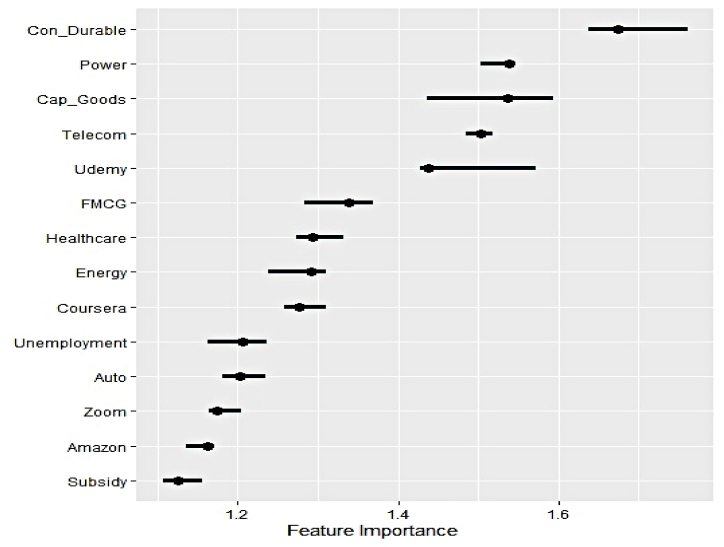
Global feature ranking for renewable electricity generation.

Fig. 8, Fig. 9, Fig. 10 display global feature ranking for explaining the variability of the electricity generation from three different sources through the lens of permutation feature evaluation.

It can be noticed that both Google search trends and sectoral market indicators have emerged to feature in the top four important features to track hydro electricity generation. Thus, dependence on household and industrial demands emerges to be equal. Predictive prowess of Google search trends on Subsidy, Unemployment transpires to be comparatively lower. Interestingly, the impact of search trends linked to strict academic programs in the form of online education manifested by Coursera and Udemy appears to be reciprocal in nature. Zoom, on the other hand, can be used in academic and official engagements simultaneously.

The dominance of sectoral outlook over Google search trends in exerting predictive influence on nuclear electricity generation is apparent. The four most significant characteristics are closing prices of the Power, Telecom, and Energy sectors. Likewise, the subdued impact of Google search trends, Udemy, Unemployment, and Subsidy, can be observed in the previous scenario. Hence nuclear electricity is primarily consumed in industrial production mainly.

Clear supremacy of the influence of industrial demand on renewable electricity over household demand is evident as the former category of explanatory features occupies the top four important feature list. Similar to nuclear electricity demand, the reliance of the telecom and power sector on renewable counterparts is evident as well. The impact of Google search trends on Udemy intensifies in explaining the variation of the underlying series, while Subsidy remains relatively less important. Overall, the permutation feature evaluation reveals critical findings in identifying the sectors that significantly absorb clean electricity and gauging the impact of household demand. As reflected by the corresponding search trend, initiatives on government subsidies have minimal effect on any form of clean electricity generation.

#### 5.3.2 Outcome of ALE-based feature evaluation

**Fig. 11:**
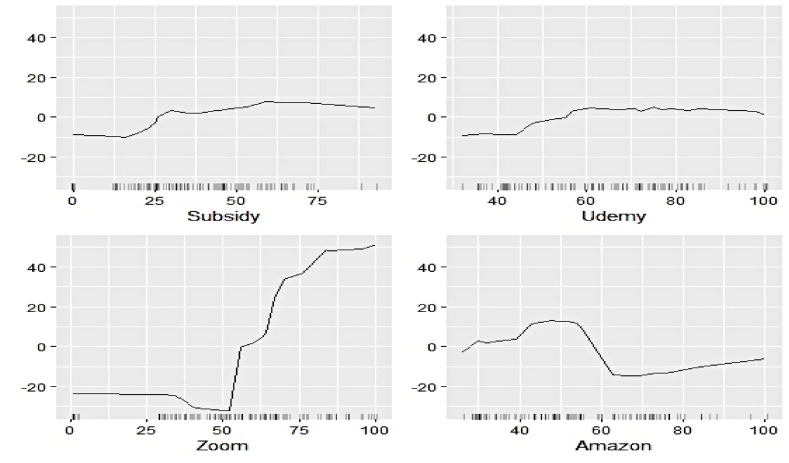
Ale plots of feature influence for hydro electricity generation (segment 1).

**Fig. 12:**
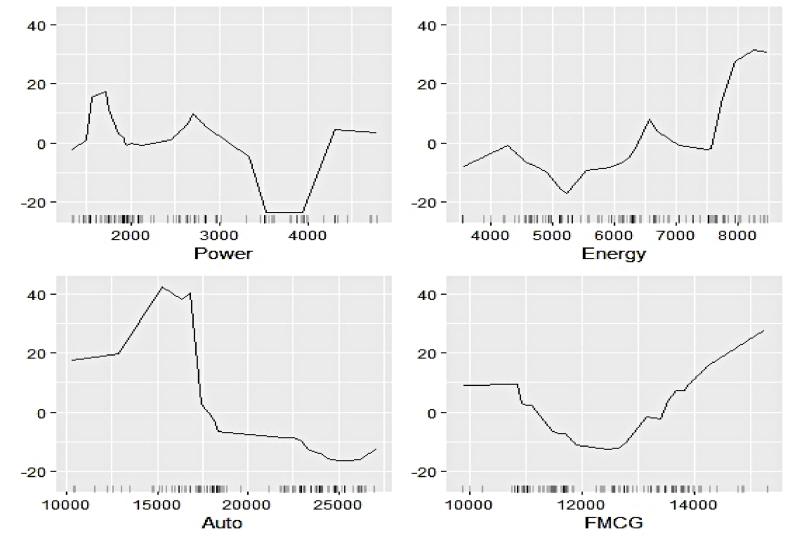
Ale plots of feature influence for hydro electricity generation (segment 2).

**Fig. 13:**
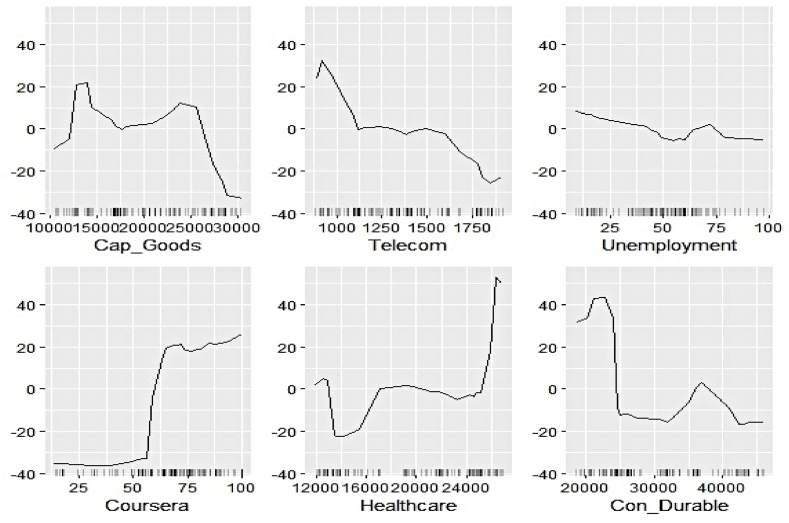
Ale plots of feature influence for hydro electricity generation (segment 3).

**Fig. 14:**
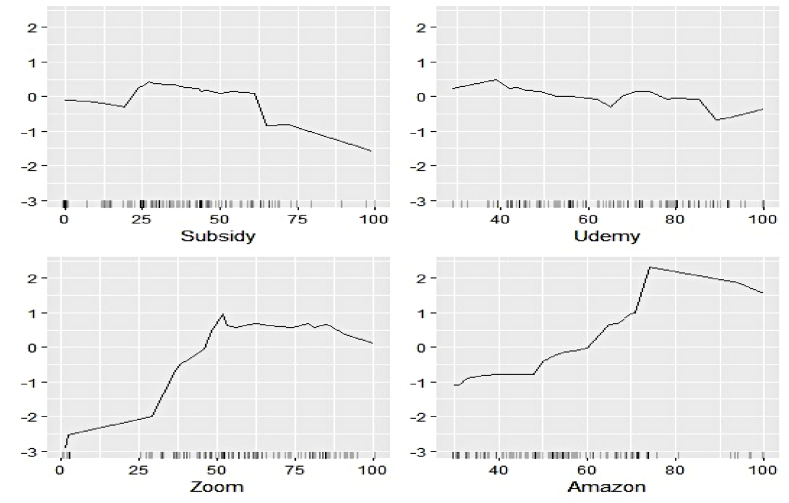
Ale plots of feature influence for nuclear electricity generation (segment 1).

**Fig. 15:**
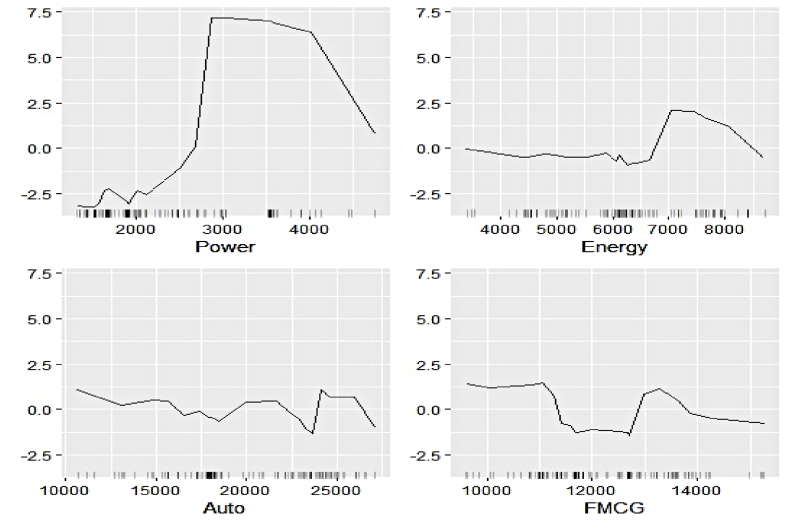
Ale plots of feature influence for nuclear electricity generation (segment 2).

**Fig. 16:**
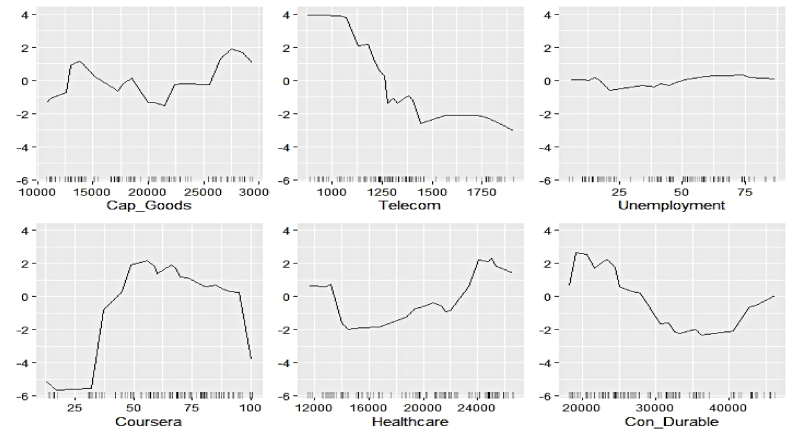
Ale plots of feature influence for nuclear electricity generation (segment 3).

**Fig. 17:**
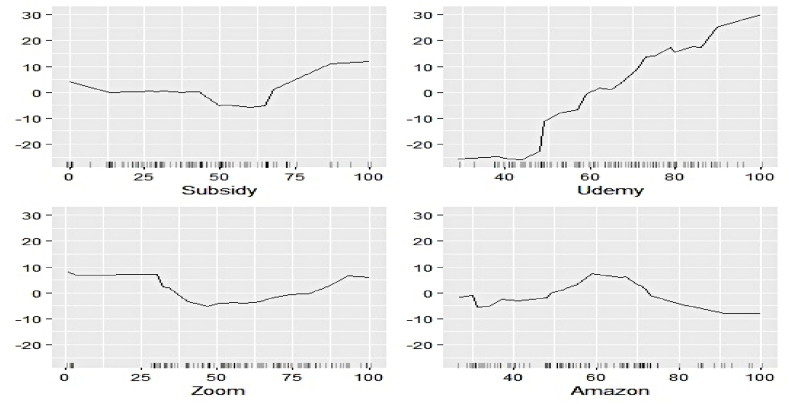
Ale plots of feature influence for renewable electricity generation (segment 1).

**Fig. 18:**
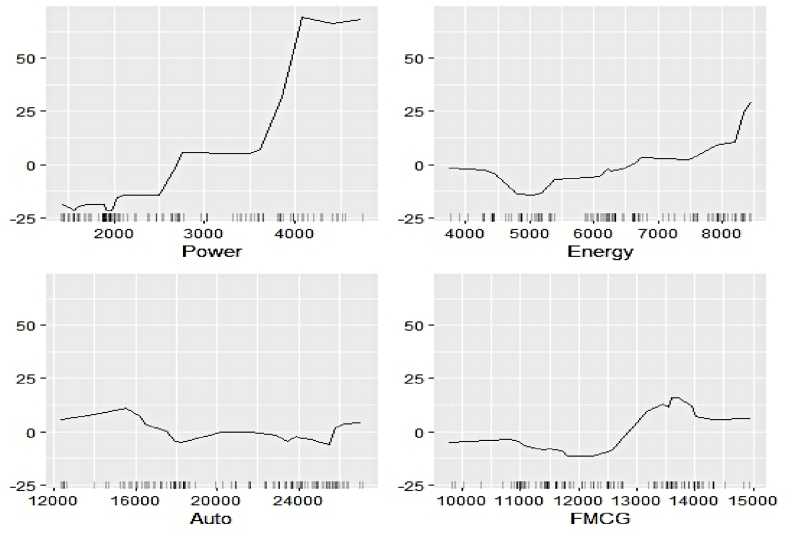
Ale plots of feature influence for renewable electricity generation (segment 2).

**Fig. 19:**
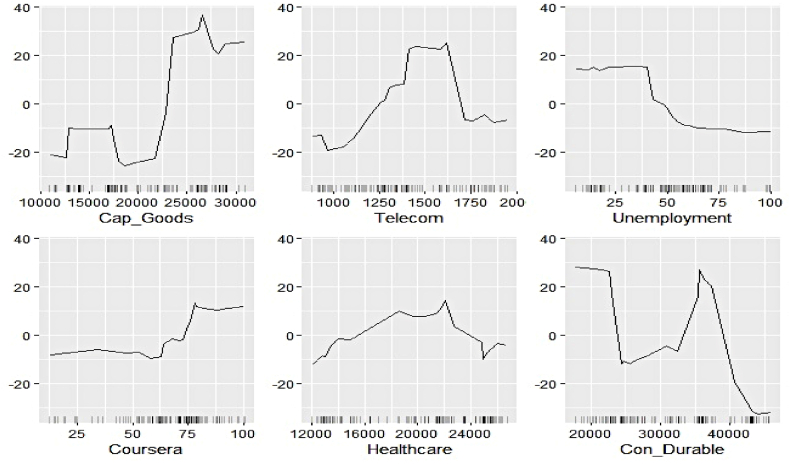
Ale plots of feature influence for renewable electricity generation (segment 3).

We now estimate the ALEs of individual features to explain their contribution to the respective electricity generation series at a more profound scale. Fig. 11, Fig. 12, Fig. 13, Fig. 14, Fig. 15, Fig. 16, Fig. 17, Fig. 18, Fig. 19 represent a visual depiction of the findings. The horizontal axis annotates the values of the respective features, while the vertical axis denotes the contribution values.

The above ALE plots of respective determinants of hydro electricity generation uncover the individual dependence structure. Contribution of Subsidy and Udemy remains flat throughout their range, conforming to the outcome of permutation feature evaluation. The impact of Zoom and Coursera increases as a steep jump in contributions can be observed after they cross threshold figures. It basically indicates longer engagement in online education sharpens the electricity appetite of the household. Amongst the industrial sector, a bullish phase was observed in Energy, FMCG, and Healthcare linked to higher hydro electricity generation. Telecom and Con_Durable, nonetheless, exhibit opposite behavior.

The ALE exhibits of determinants of nuclear electricity generation unveil interesting insights too. The magnitude of influence of the Google search indicators can be seen to be relatively lower than that of the sectoral ones. Thus, the domination of the industrial demand over the household over nuclear electricity generation, as observed in permutation feature evaluation, is validated. The impact of several features remains stagnant over different intervals. On the other hand, decreasing predictive power of Telecom, Subsidy, and FMCG with an increase of their respective values can be noticed.

The ALE plots of renewable electricity generation suggest a strong positive influence on Power and Udemy as their values cross a threshold. The influence of Unemployment diminishes as its search pattern intensifies. A spike in the contribution of Coursera bounded to a specific interval is apparent. No substantial difference in the predictive contribution of the remaining search indicators could be found, as ALE plots do not document sharp increases or decreases. As deemed critical in the permutation feature evaluation, Power, Cap_Goods, and Telecom are linked to increasing positive influence, whereas Con_Durable exerts negative predictive power.

In this nutshell, the utilization of the permutation feature evaluation and ALE plots simultaneously caters to the proper interpretation of functionalities of the underlying explanatory variables globally. The key contributory features have been discovered in conjunction with explaining the dependence structure.

#### 5.3.3 Outcome of LIME-based feature evaluation

**Fig. 20:**
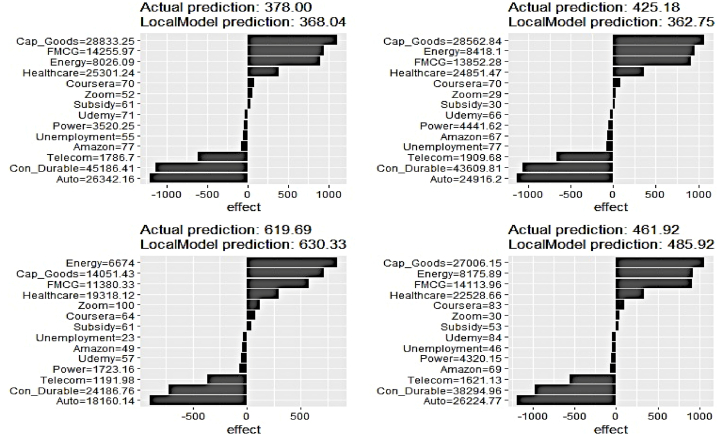
LIME Analysis of Hydro Elect. Generation on 4 randomly chosen data samples.

**Fig. 21:**
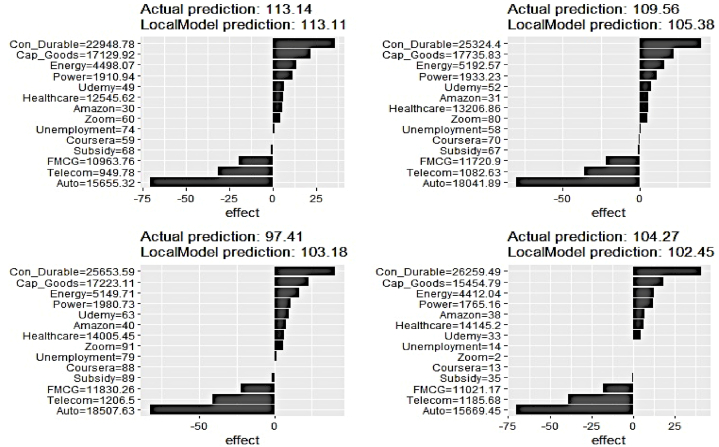
LIME Analysis of Nuclear Elect. Generation on 4 randomly chosen data samples.

**Fig. 22:**
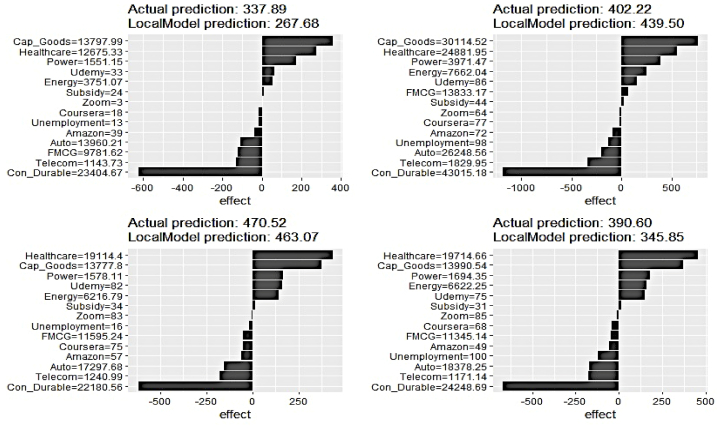
LIME Analysis of Renewable Elect. Generat. on 4 randomly chosen data samples.

We, now, proceed to delve into the prediction process at the local level applying the LIME plots on four randomly chosen data samples for select clean electricity generation series. Fig. 20, Fig. 21, Fig. 22 depict the outcome. The vertical axis annotates the explanatory features, while the horizontal axis represents their respective contributions.

It is amply apparent that the local level feature ranking for predicting hydro electricity generation is not uniform across the samples and substantially differs from the global ranking. The contribution of Subsidy and Udemy is no longer negligible to drive prediction on the chosen samples. Cap_Goods, Energy, FMCG, and Healthcare occupy the top four important feature spots with different orders of ranking across the samples.

Similar to the previous case, local-level feature contribution has emerged to be different from that of the global level. No Google search indicators feature in the top four important feature lists in driving predictions on the select data samples. The influence of Telecom has seen a substantial dip in the select samples, whereas the effects of Udemy have increased.

The outcome of the local feature evaluation for renewable electricity generation has demonstrated similarity to its counterparts as a significant difference in the contribution pattern to that of the global scale is imminent. Interestingly, Udemy has featured in the top four feature list in two samples, wherein no search indicators were deemed to be highly important in explaining the overall variability of the renewable electricity generation pattern. The predictive prowess of the Con_Durable has experienced a drastic reduction for the selected data instances.

Therefore, the overall assessment through the ALE plots underscores the utility of all underlying features in precisely tracking the clean electricity generation pattern. It is equally important to emphasize explaining the short-run fluctuation owing to practical implications. As the current work is the first of its kind, the inclusion of Google search trend indicators for making is unique. Although on a global scale, domination of the sectoral indices reflecting industrial growth and demand has relatively outperformed the search indices in terms of predictive prowess, the utility of the latter is proven in the local-level prediction processes. Thus, the findings of XAI methodologies also rationalize the selection of explanatory features.

## 6 Discussion

The findings of the present research echo that the chosen daily electricity generation from the chosen renewable sources is highly predictable, implying steady growth and reliance on the same for household and industrial activities in the Indian context. Initially, the daily electricity generation series from the chosen sources has been found to follow a long-memory dependence structure. Hence, the demand for the same can be inferred to exhibit high volatile phases followed by high volatile regimes and low volatile phases followed by low volatile regimes. Thus, the eventual consumption patterns are unlikely to experience sporadic movements, which can be used for regulatory frameworks. The hydro electricity generation has been observed to be relatively more predictable, which is followed by nuclear and renewable electricity generation. The strong predictive influence of selected socio-economic factors manifested by the financial outlook of Indian sectoral indices and Google search trend indicators suggest tracking daily clean electricity demand can be facilitated by gauging industrial production and user engagement in various activities. The prosperity of Edtech companies, viz. Coursera, Udemy, etc., have garnered high traffic, which indirectly catalyzed the transition toward clean electricity owing to increased reliance on computational power. On the other hand, different industrial sectors do not spur daily clean electricity generation uniformly, as revealed by the outcome of XAI-based modeling.

The other aspect of the contribution, i.e., the propounded forecasting structure, is of immense practical implications as the same survives a series of numerical and statistical tests. The robustness of the framework is apparent as the dynamics can be explained precisely during the COVID-19 pandemic and Russia-Ukraine conflict regimes. The efficiency of the framework in minutely estimating the directional changes as manifested by high DA figures on both training and test segments has been established. The said characteristics of the framework could be suitably used to anticipate peak or fall in demand in short-run horizons, enabling effective resource planning at power plants. Accurate estimation of absolute figures and directional changes is of paramount relevance for risk management in uncertain periods. The success of the ASO metaheuristic algorithm in augmenting the predictive performance of the proposed granular forecasting structure is evident, which significantly contributes to the superiority of the proposed model over benchmark competing methodologies. The property handling nonlinear and nonstationary time series augments the potential of the ASO-based EEMD-DBSCAN granular approach. Overall, the effectiveness of the methodological framework can easily be extended to carry out predictive modeling of financial markets, wherein the stakes are even higher.

The scope of underlying research is restricted to three clean electricity resources in the Indian context with chosen macro indicators as explanatory variables. As stated, the previous research has primarily been carried out in micro setups to gauge power generation or consumption patterns. Hence, the research findings are useful to mitigate the demand and supply gap at the country level and strategize increased adoption of clean electricity. Nevertheless, the spectrum of the research can easily be extended into cross-country comparisons of clean electricity production processes to gauge whether the degree of predictability differs across developed and developing economies. It is also possible to introspect the behavioral pattern in different regions of a country in a state-wise manner for deeper inspection. A few micro-process-specific variables can be incorporated into the research framework to explain the leftover variability of chosen time series. Comparative modeling of daily nonrenewable and clean electricity generation in developed and developing economies can be carried out to deeply comprehend the consumption dynamics and influence of cognate macroeconomic and other factors. The capacity of the predictive framework can be utilized for analyzing complex financial time series during Black Swan events. On the methodology front, a comparison of the capability of state-of-the-art deep learning algorithms with ensemble machine learning techniques in deriving predictions through the ASO-based granular forecasting framework can also be examined in the future.

## 7 Conclusions

The underlying research endeavors to critically analyze the pattern of clean electricity generation in the Indian context through the applied predictive analysis lens. We advance a novel EEMD-DBSCAN-based decomposition methodology and integrate the same with the ASO-based EoE forecasting structure to delve into the predictability of hydro, nuclear, and clean electricity generation patterns for accomplishing the objectives. Additionally, the current work strives to uncover the deeper insights pertinent to the predictive influence of the industrial and household demand governing clean electricity growth.

The overall findings of the present research suggest that the daily clean electricity generation in India from hydro, nuclear, and renewable sources can indeed be predicted with a high degree of accuracy. The utility of utilizing socio-economic factors by incorporating Google search indicators and sectoral outlook as explanatory variables for tracking clean electricity generation is established. The study is highly relevant for maintaining sustainability and reducing carbon emissions. The values of the DA indicator for the prediction of the three energy generation series have emerged to be higher than 0.9, which signifies the effectiveness of the research framework in accurately determining the immediate directional changes in electricity generation. The quality of forecasts on both setups, 70%–30 % and 85%–15 % covering chaotic and volatile regimes testify to the validity of the model. The designed granular structure has transpired to yield statistically superior forecasts over 4 competing models, which rationalizes its relative efficiency. We critically delve into the predictive interplay of hydro, nuclear, and renewable electricity generation with important socio-economic factors by incorporating Google search indicators and sectoral outlook. The global and local level feature interpretation reflects the nature and importance of the explanatory variables. Among the sectors, Cap_Goods, Con_Durable, Power, and Energy have emerged to drive clean electricity growth in India prudently. It, nonetheless, is necessary to monitor the growth and prosperity of other select sectors as well as to explain the abrupt variation of clean electricity demand, which can streamline the generation process in the long run. The relatively better predictability of hydro electricity generation over the other two counterparts implies a stable intake of the same in running a business and day-to-day activities. The accuracy of the predictions, specifically during the COVID-19 regime's emphasis on clean electricity generation in India, remained less perturbed despite heavy disruptions and lockdowns. From a policy-making perspective, providing incentives or rolling out appropriate schemes to migrate to hydro, nuclear, and renewable power for sectors that are linked to comparatively low dependence on the same can be chalked out. In the Indian context, fear of unemployment and the tendency to avail subsidy offerings have emerged not to be critical in shaping the clean electricity generation process, indicating a presence of reasonably effective governance. Industrial production predominantly consumes nuclear and renewable electricity. Nevertheless, the short-run electricity appetite of household operations at the individual level is apparent too.

The present work significantly contributes to the methodological front for conducting predictive analysis of clean electricity generation. Our work propounds a robust granular predictive framework that not only produces highly accurate forecasts but scrupulously survives validation and comparative performance assessments. Utilizing the DBSCAN-based clustering framework for disentangling the original series into high and low-frequency subseries immensely facilitates the training process. Subsequently, the seamless integration of three ensemble machine learning models applying the ASO-based optimization setup significantly improves the predictive accuracy. The framework is, therefore, classified as a significant addition to the granular methodological spectrum. The profound dependence of clean electricity generation on both sets of factors has been established, which explains the apparently nonlinear and nonstationary pattern. The practical implications of the predictive framework in facilitating energy trading further underscore the contribution.

## Funding

This research was funded by 10.13039/501100007480University of Castilla-La Mancha, grant number [2022- GRIN-34255] and Regional Government of Castilla-La Mancha, grant number [SBPLY/21/180225/000110].

## Data availability statement

Data associated with our study has not been deposited into a publicly available repository but will be made available on request.

## CRediT authorship contribution statement

**Indranil Ghosh:** Writing – original draft, Software, Methodology, Conceptualization. **Esteban Alfaro-Cortés:** Visualization, Project administration, Investigation, Data curation. **Matías Gámez:** Writing – original draft, Supervision, Software, Funding acquisition. **Noelia García-Rubio:** Writing – review & editing, Validation, Investigation, Formal analysis.

## Declaration of competing interest

The authors have no conflicts of interest to declare. All co-authors have seen and agree with the contents of the manuscript and there is no financial interest to report. We certify that the submission is original work and is not under review at any other publication.
